# Evaluation of in-house PCR for diagnosis of smear-negative pulmonary tuberculosis in Kampala, Uganda

**DOI:** 10.1186/1756-0500-5-487

**Published:** 2012-09-05

**Authors:** Lydia Nakiyingi, David P Kateete, Ponsiano Ocama, William Worodria, Joseph B Sempa, Benon B Asiimwe, Fred A Katabazi, Achilles Katamba, Laurence Huang, Moses L Joloba, Harriet Mayanja-Kizza

**Affiliations:** 1 Infectious Diseases Institute, Makerere University College of Health Sciences, Mulago Hospital Complex, Kampala, Uganda; 2 Department of Medicine, School of Medicine, Makerere University College of Health Sciences, Kampala, Uganda; 3 Department of Medical Microbiology, School of Biomedical Sciences, Makerere University College of Health Sciences, Kampala, Uganda; 4 HIV/AIDS Division and Division of Pulmonary and Critical Care Medicine, San Francisco General Hospital, University of California-San Francisco, San Francisco, CA, USA

**Keywords:** Pulmonary tuberculosis, Smear-negative TB, HIV-infected, HIV-TB co-infection, CD4 cell counts, Nucleic acid amplification tests, In-house PCR, Lowenstein-Jensen culture, Sensitivity, Specificity, Resource limited settings

## Abstract

**Background:**

Nucleic acid amplification tests (NAATs) have offered hope for rapid diagnosis of tuberculosis (TB). However, their efficiency with smear-negative samples has not been widely studied in low income settings. Here, we evaluated in-house PCR assay for diagnosis of smear-negative TB using Lowenstein-Jensen (LJ) culture as the baseline test. Two hundred and five pulmonary TB (PTB) suspects with smear-negative sputum samples, admitted on a short stay emergency ward at Mulago Hospital in Kampala, Uganda, were enrolled. Two smear-negative sputum samples were obtained from each PTB suspect and processed simultaneously for identification of MTBC using in-house PCR and LJ culture.

**Results:**

Seventy two PTB suspects (35%, 72/205) were LJ culture positive while 128 (62.4%, 128/205) were PCR-positive. The sensitivity and specificity of in-house PCR for diagnosis of smear-negative PTB were 75% (95% CI 62.6-85.0) and 35.9% (95% CI 27.2-45.3), respectively. The positive and negative predictive values were 39% (95% CI 30.4-48.2) and 72.4% (95% CI 59.1-83.3), respectively, while the positive and negative likelihood ratios were 1.17 (95% CI 0.96-1.42) and 0.70 (95% CI 0.43-1.14), respectively. One hundred and seventeen LJ culture-negative suspects (75 PCR-positive and 42 PCR-negative) were enrolled for follow-up at 2 months. Of the PCR-positive suspects, 45 (60%, 45/75) were still alive, of whom 29 (64.4%, 29/45) returned for the follow-up visit; 15 (20%, 15/75) suspects died while another 15 (20%, 15/75) were lost to follow-up**.** Of the 42 PCR-negative suspects, 22 (52.4%, 22/42) were still alive, of whom 16 (72.7%, 16/22) returned for follow-up; 11 (26.2%, 11/42) died while nine (21.4%, 9/42) were lost to follow-up. Overall, more PCR-positive suspects were diagnosed with PTB during follow-up visits but the difference was not statistically significant (27.6%, 8/29 vs. 25%, 4/16, *p* = 0.9239). Furthermore, mortality was higher for the PCR-negative suspects but the difference was also not statistically significant (26.2% vs. 20% *p* = 0.7094).

**Conclusion:**

In-house PCR correlates poorly with LJ culture for diagnosis of smear-negative PTB. Therefore, in-house PCR may not be adopted as an alternative to LJ culture.

## Background

The genetically homogeneous subspecies of the *Mycobacterium tuberculosis* complex (MTBC; *M. tuberculosis, M. bovis, M. bovis* BCG, *M. africanum, M. caprae* and *M. cannetti*) cause tuberculosis (TB) [1,2], a global disease that affects one third of the human population [3,4]. TB and HIV co-infection affects many in sub-Saharan Africa [5-7]; Uganda has a high HIV prevalence and is also among the world’s 22 high TB-burdened countries with an estimated incidence of 402 cases per 100,000 individuals [3]. Kampala, the capital of Uganda has approx. 2 million inhabitants and accounts for approx. 30% of the nation’s TB burden [4].

Accurate diagnosis is crucial for efficient management of TB patients [3]; however, TB diagnosis remains a challenge particularly in resource limited settings (RLS) where the disease is complicated by HIV co-infection.

Conventional approaches to TB diagnosis in RLS still rely on methods that have major limitations [8-10]. Smear microscopy is the most widely available method but has varying sensitivity (30 to 60%) particularly in TB-HIV co-infected patients. The chest X-ray, often a supplementary test for diagnosis of smear-negative pulmonary TB (PTB) also has low specificity. Solid cultures are used as confirmatory tests but are expensive, lengthy (up to 8 weeks) and not widely available in RLS [11].

The World Health Organization (WHO) recommends liquid cultures in high TB burdened countries due to the advantage of rapid detection and incremental yield in comparison with solid media [12]. However, liquid culture systems are expensive, prone to contamination and usually support the growth of non-tuberculous mycobacteria (NTM).

Nucleic acid amplification tests (NAATs) are promising new methods for rapid detection of *M. tuberculosis* (MTB) directly in samples or TB culture and are being considered as cost-effective alternatives in RLS [13,14]. The latest development was the WHO’s endorsement of the GeneXpert (Xpert MTB/Rif) for use in TB endemic countries, declaring the system a major milestone for global TB diagnosis. The high cost notwithstanding [15], some sub-Saharan African countries (e.g. South Africa, Morocco, etc.) have introduced the Xpert MTB/Rif system for routine TB diagnostics. Even then, research on the optimal use of NAATs for TB diagnosis is still wanting in sub-Saharan Africa where there is high burden of HIV/TB co-infection.

An in-house PCR assay for rapid identification of MTBC in smear-positive sputum samples and acid fast bacilli (AFB) positive cultures was previously introduced in this setting [16], but it has never been evaluated for the diagnosis of smear-negative PTB in the same setting. Using LJ culture as the base-line test, this study evaluated in-house PCR for rapid diagnosis of smear-negative PTB in a low income setting with high burden of TB/HIV co-infection.

## Methods

### Setting, participants and specimen collection

This study was conducted between September 2007 to February 2008, on a short stay emergency medical ward at Mulago National Referral and Teaching Hospital in Kampala, Uganda. The emergency ward temporarily admits and triages patients before transfer to specialized medical units. Approx. 30 patients per day are admitted, of whom one third have respiratory symptoms. Patients with respiratory symptoms were examined by specialists who identified PTB suspects. PTB suspects were defined as patients with cough for ≥2 weeks with or without any of the following; sputum production, haemoptysis, chest pain, shortness of breath, loss of appetite, weight loss, fatigue, night sweat and fever.

Two sputum samples (one on spot and another early morning) were collected from each PTB suspect and examined by Ziehl-Neelson (ZN) microscopy for identification of AFB [17]. Sputum induction (using 7% hypertonic saline inhaled by nebulization) was used for patients who were unable to expectorate sputum.

### Inclusion/exclusion criteria

**Figure 1:**
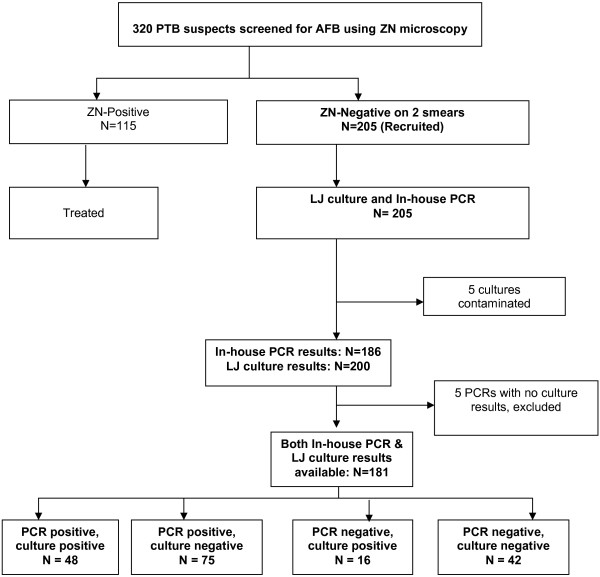
Study flow chart.

A total 320 PTB suspects were screened; patients with smear-positive sputum samples were excluded but started on TB treatment according to the Uganda National TB guidelines. To be enrolled in the study, a PTB suspect ought to have consecutively produced at least two AFB smear-negative sputum samples upon ZN microscopy. Overall, sputum samples for culture and DNA extraction were obtained from a total of 205 PTB suspects who met the above criteria (i.e. two smear-negative sputum samples); these were recruited as study participants (Figure 1). In addition, demographic and clinical data were obtained from the 205 enrolled participants.

### HIV testing

HIV testing was performed for all the enrolled patients following the algorithm for the ministry of health, Uganda [18]. CD4+ cell counts by BD FACS calibur (Becton and Dickinson, Franklin Lakes, NJ, USA) were performed, as well as chest X-rays.

### Sputum processing

Sputum processing and culture were performed in biosafety level 3 facility at the national TB reference laboratory (NTRL) in Kampala, Uganda. The sputum samples were processed by digestion and decontamination in a bio-safety cabinet class II as previously described [16,19]. Briefly, 200 μl of digestion buffer (2.9% sodium citrate, N-Acetyl L-cysteine [NALC] and 6% NaOH) was added to an equal volume of sputum, vortexed and incubated at room temperature for 15 min. The digested sample was then diluted to 50 ml with phosphate buffer (pH 6.8), mixed thoroughly and centrifuged at 4000 *g* for 15 min; the sediment was then suspended in 2 ml phosphate buffer.

### MTBC cultures

LJ culture, widely used for TB diagnosis in RLS [20], was used as a baseline test to assess the diagnostic accuracy of in-house PCR. LJ culture was chosen as the gold standard because all the mycobacterial colonies on LJ-positive samples in this setting are predominantly MTBC [21,22]. For cultures, 100 μl each of the processed sputum (see above) was inoculated into LJ culture bottles and incubated at 37°C for up to 3 months. Cultures were considered positive only if mycobacterial colonies appeared within 8 weeks following inoculation. Colonies from culture-positive LJ bottles were confirmed for presence of AFB by ZN microscopy and 16 s rRNA PCR.

16S rRNA PCR was performed on LJ-positive cultures to confirm mycobacteria (which were presumptively regarded MTBC [21,22]) and rule out subtle growth from other acid fast bacilli organisms on LJ media (such as *Nocardia*, Corynebacteria and Frankia). PCRs were performed on all LJ-positive cultures using the following primers: 5'-ACG GTG GGT ACT AGG TGT GGG TTT C-3', forward and 5'-TCT GCG ATT ACT AGC GAC TCC GAC TTC A-3', reverse. The amplification program was as follows: initial denaturation at 94°C for 4 min, followed by 31 cycles each consisting of denaturation at 94°C for 30s; annealing at 63°C for 30s and extension at 72°C for 45 s. Then, there was a final extension at 72°C, for 10 min. Amplicons were analyzed by agarose gel electrophoresis in which a 600 bp fragment was detected in positive samples.

### Chromosomal DNA extraction

DNA extraction and molecular assays were performed at the Molecular Biology Laboratory, Department of Medical Microbiology, Makerere University College of Health Sciences. Approx. 0.5 ml each of the processed sputum samples (see sputum processing) in screw-capped cryovials (Nalgene, Thermo Fisher Scientific, Rochester, USA) were incubated at 80°C for 2 h to heat kill the bacilli. Then, chromosomal DNA was extracted with the Master pure™ purification kit (Epicentre Biotechnologies, Madison, USA) following the manufacturer’s guidelines.

### In-house PCR assays

The IS6110 insertion sequence, which is unique to the MTBC members [23,24] was the target for the in-house PCR assay. Amplification reactions were performed with primers, P43 (forward, 5'-TCAGCCGCGTCCACGCCGCCA-3'), and P53 (Reverse, 5'-CCGACCGCTCCGACCGACGGT-3') [16], which amplify 521 bp of IS6110. Each reaction contained 20 pmoles of the forward and reverse primer, 1 μl of custom PCR-Master mix (10 mM Tris-HC1, pH 9.0, 2 mM MgCl_2_, 50 mM KCl, 200 μM dNTPs and 5% DMSO), 0.5U *Taq* polymerase and 2 μl of chromosomal DNA template in a reaction volume of 10 μl.

Amplifications were performed in the PTC-200 Peltier thermocycler (MJ Research, Waltham, USA) under the following conditions: initial denaturation at 94°C for 5 min; followed by 34 cycles each consisting of 94°C, 30 s; 65°C, 30 s; and 72°C, 45 s; and a final extension step at 72°C for 10 min. Then, amplicons were electrophoretically analyzed using 1% agarose gel in TBE (Tris-Borate EDTA) buffer stained with ethidium bromide and visualized under ultraviolet (UV) light in a UV transilluminator. Positive control reactions included template DNA purified from MTB strain H_37_Rv, while negative controls included reactions with only pure nuclease free water or DNA extracted from *M. smegmatis* and *Escherichia coli*. Presence of an approx. 500 bp fragment in the test lanes indicated presence of MTBC in the sample provided controls were valid.

### Patient follow-up

To determine survival status and confirm diagnosis of TB, follow-up at 2 months (window 8 to 16 weeks) was done for PCR-positive/culture negative as well as PCR-negative/culture negative study participants and the outcomes of both categories compared. ZN-sputum microscopy was performed during the follow-up visits. Additionally, medical records and additional test-results were also reviewed. Information was obtained by telephone interviews for participants who were unable to return for follow-up visits. Patients were classified as having PTB based on any of the following**:** MTBC isolated in at least one culture; positive ZN sputum smear; granulomas on histopathology; and clinical response to TB treatment in absence of a non-TB alternative diagnosis.

### Quality control

Cross contamination of cultures was minimized through use of sterile disposable aerosol resistant tips. For each sample, separate tubes with decontamination/phosphate buffers were used to avoid cross-transfer of specimens. Samples with only phosphate buffer were always included in the batch being processed and these remained negative upon culture. For molecular assays, separate rooms were used for sample preparation, reaction mixes, DNA amplification and detection. After use, UV hoods were decontaminated by turning on UV light. Negative controls were included in each PCR batch to detect cross-contamination during premixes. To determine the effect of PCR inhibitors, reactions were spiked with 500 ng of MTB chromosomal DNA from the reference strain H_37_Rv (ATCC 27294) and ran in parallel; amplification of the IS6110 fragment implied absence of or minimal PCR inhibition. All laboratory personnel were blinded to the clinical and culture data.

### Data analysis

The data were analyzed with STATA version 10.0 (StataCorp LP, College Station, TX, USA). The sensitivity, specificity, positive and negative predictive values as well as diagnostic likelihood ratios for in-house PCR assay were calculated using LJ culture as the base line test. To compare clinical outcomes (TB diagnosis and mortality) at 2 months of follow-up between PCR-positive/culture negative and PCR-negative/culture negative participants, a Z-test was used to test for differences in proportions. A *p* value of < 0.05 was considered statistically significant.

### Ethical considerations

The study was approved by the Makerere University Faculty of Medicine Research and Ethics Committee. Written informed consent was obtained from all the patients who participated in this study.

## Results

Two hundred and five smear-negative PTB suspects were recruited, with a mean age of 34.7 years (±10.4 standard deviation) and an equal gender distribution. There were few smokers and most patients were HIV-infected (85.9%, 176/205), of whom 72.2% (127/176) had advanced immunosuppression (CD4+ cell count of ≤ 200 cells/μL). Furthermore, many patients had abnormal chest findings (76.1%, 156/205). Although many patients reported fever, only 41% (84/205) had a body temperature of  ≥ 37.5°C at enrolment (Table 1).

**Table 1:** Baseline characteristics of smear-negative PTB suspects (n = 205)

**Characteristics**	**Frequency**	**Percentage**
**Socio-demographics**		
Females	105	51.2
Never smoked	140	68.2
Smoked	65	31.8
**Clinical parameters as reported by patients**		
Cough (>2 weeks)	205	100
Fever and/or night sweats	193	94.1
Weight loss	191	93.2
Wheezing	42	20.5
Difficulty in breathing	130	63.4
Blood in sputum	57	30.1
Antibiotic exposure in previous 2 weeks	84	41.0
**Clinical examination (physical)**		
Body temperature (°C)*		
<37.5	113	57.4
≥37.5	84	42.6
≤20	45	23.6
>20	146	76.4
Oxygen saturation (% measured by a pulseoximeter)*		
≤90	47	23.1
>90	156	76.9
*Pulse rate, beats/min (measured by a pulseoximeter)*		
Median rate (percentile range)	106 (88 to119)	
*Lung examination*		
Normal (clear chest)	49	23.9
Abnormal (Rhonchi, crepitation, bronchial breathing, absent breath sounds)	156	76.1
*HIV status*		
Positive	176	85.9
Negative	29	14.1
CD4 Cell Count* (cell/ml)		
≤ 200	127	76
>200	40	24

*Some measurements missing.

Of the 205 cultured samples, 72 (35.1%) grew mycobacterial colonies on LJ media. Since LJ culture method has been found non-conducive for growth of non-tuberculous mycobacteria in our setting [21,22] all the LJ culture-positive samples were regarded as MTBC. Furthermore, 128 (62.4%) samples had no visible growth while five (2.4%) cultures were contaminated (Figure 1).

Of the 205 sputum samples analyzed by in-house PCR, 19 (9.3%, 19/205) results were not available leaving 186 (90.7%, 186/205) PCR results for analysis. Of the 186, 128 (68.8%, 128/186) were confirmed as MTBC while five (5/186) had the corresponding LJ culture results contaminated hence excluded (Figure 1).

### Performance of the in-house PCR in diagnosing smear-negative PTB

Five PCR samples had culture results contaminated and were excluded from the analysis leaving 181corresponding PCR and LJ culture results (Figure 1 and Table 2). The sensitivity and specificity of in-house PCR in diagnosing smear-negative PTB was 75% (95% CI 62.6-85.0) and 35.9% (95% CI 27.2-45.3), respectively. The positive and the negative predictive values were 39% (95% CI 30.4-48.2) and 72.4% (95% CI 59.1-83.3), respectively. The positive and negative likelihood ratios were 1.17, 95% CI (0.96-1.42) and 0.7, 95% CI (0.43-1.14) respectively. Details of these performance indices are shown in Table 2 and 3.

**Table 2:** Performance indices for In-house PCR using LJ Culture as the base line test

	***Culture Positive***	***Culture Negative***	**Total**
*PCR Positive*	48	75	123
*PCR Negative*	16	42	58
**Total**	64	117	181

**Table 3:** More performance indices for In-house PCR

**Performance Indices**	**Index value**	**95% CI**
Sensitivity	75%	62.6-85.0
Specificity	35.9%	27.2-45.3
Positive Predictive Value	39%	30.4-48.2
Negative Predictive Value	72.4%	59.1-83.3
Diagnostic Likelihood ratio (Positive)	1.17	0.96-1.42
Diagnostic Likelihood ratio (Negative)	0.70	0.43-1.14

### Clinical outcomes

**Figure 2:**
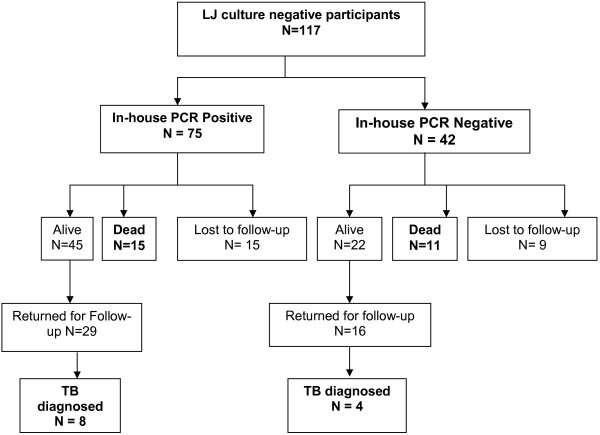
Clinical outcomes for LJ-culture negative patients comparing PCR-positive and PCR-negative groups at 2 months follow-up.

One hundred and seventeen culture-negative suspects (75 PCR-positive and 42 PCR-negative) were enrolled for the 2 months follow-up visit. Of the 75 PCR-positive ones, 45 (60%) were still alive of whom 29 (64.4%, 29/45) returned for the follow-up visit; 15 (20%) suspects died while another 15 (20%) were lost to follow-up (Figure 2). Of the 42 PCR-negative suspects, 22 (52.4%) were still alive of whom 16 (72.7%, 16/22) returned for follow-up (Figure 2); 11 (26.2%, 11/42) suspects died while nine (21.4%, 9/42) were lost to follow-up.

Overall, more PCR-positive suspects were diagnosed with TB during follow-up but the difference was not statistically significant (27.6%, 8/29 vs. 25%, 4/16, *p* = 0.9239). On the other hand, mortality was higher for PCR-negative suspects but the difference was also not statistically significant (26.2% vs. 20% *p* = 0.7094).

## Discussion

In this study, in-house PCR correlated poorly with LJ culture for diagnosis of smear-negative PTB. Although reported previously in other settings [25,26], this is among the few studies evaluating the performance of in-house PCR on smear-negative PTB suspects in Uganda, a low income country with high rates of HIV/TB co-infection. The sensitivity for the in-house PCR in the current study was higher than that reported in an earlier African study on ZN-negative sputum samples (i.e., 75% vs. 40%) [26]. Although we endeavored to control for PCR inhibitors, the sensitivity of the DNA polymerase can be affected by the paucibacillary nature of specimens [27], which could also have affected the PCR assay sensitivity in our study.

Although the performance indices in this study were estimated with LJ culture which is not the gold standard for MTBC identification, virtually all LJ-positive cultures in this setting are MTBC and speciation tests to confirm MTB are deemed unnecessary since colonies are presumptively MTBC [22]. LJ culture also is widely used as a gold standard for TB diagnosis in RLS [20].

Since our concern was mostly TB-diagnosis (i.e. patient-care) of which LJ is the gold standard for MTBC culture, we thus did not speciate cultures but confirmed the presumptive MTBC as mycobacteria through 16S rRNA PCR.

Specificity for the in-house PCR in the current study was also low (35.9%). Although a couple of PCR studies have achieved high specificity with smear-negative PTB, they mostly worked with commercial tests [28,29] that are expensive for many in RLS. Otherwise, most studies with in-house PCR on smear-negative PTB have revealed substantial variability in specificity [28,30].

It is still possible that the many false positives in the current study could have resulted from the low sensitivity of LJ culture [22,31]. Indeed, the culture-positive/PCR-negative isolates could have been NTM, which are known to cause severe disease in immunocompromised HIV-positive patients with low CD4+ counts. Moreover, majority of the subjects in this study were HIV-infected with low CD4+ cell counts. We hope future studies will consider these omissions (i.e. speciating NTM among AFB smear-negative PTB suspects).

Furthermore, while the Flores et al. 2005, meta-analysis for in-house PCR accuracy [28] found the IS6110 amplification target highly accurate, this was not shown in the current study, meaning that IS6110 alone may not be adequate for increased diagnostic yield. The diagnostic likelihood ratio (DLR) for a positive in-house PCR was 1.17 [95% CI (0.96-1.42)] implying that a positive in-house PCR test may not indicate presence of MTBC. Likewise, the negative DLR was 0.7 [95% CI (0.43-1.14)] implying that a negative in-house PCR test is not indicative of absence of MTBC. Therefore, with in-house PCR in this setting, a clinician will need additional diagnostic methods to confirm PTB in smear-negative suspects.

There was no significant difference in mortality and diagnosis of TB at follow-up between PCR-positive/culture negative and PCR-negative/culture negative PTB suspects, although mortality was higher for PCR-negative suspects. However, this could be due to other factors that were not addressed, for instance co-morbidities.

### Limitations

Due to limited funding, we did not use biochemical tests or DNA sequencing which methods are considered gold standards for MTBC identification; probably these would have provided higher accuracy estimates for the in-house PCR. Species-confirmation of the LJ-positive cultures was not done in light of recent findings in parallel studies in this setting, in which AFB growth on LJ medium is virtually MTBC [21,22].

Few participants returned for the follow-up visits and we were unable to establish the possible cause of death in the participants who died. Although predictive values are reported, these cannot be accurately interpreted in this pooled population. Lastly, this study does not represent the general use of in-house PCR in a real world setting, since PCR methods vary widely with setting and the data herein may not be generalizable.

## Conclusions

In-house PCR is inefficient for diagnosis of smear-negative PTB. Its diagnostic accuracy is low and it may not be used as an alternative for LJ culture in this setting.

## Abbreviations

AFB: Acid fast bacilli; BSL-3: Biosafety level 3; DMSO: Dimethyl sulfoxide; dNTPs: Deoxyribonucleotide triphosphates; EDTA: Ethylenediaminetetraacetic acid; ELISA: Enzyme linked immunosorbent assays; HIV: Human immunodeficiency virus; LJ: Lowenstein-Jensen media; MTB: *Mycobacterium tuberculosis*; MTBC: *Mycobacterium tuberculosis* complex; NAAT: Nucleic acid amplification tests; NALC: N-Acetyl L-cysteine; NPV: Negative predictive value; NTRL: National tuberculosis reference laboratory; NTM: Non tuberculous mycobacteria; PCR: Polymerase chain reaction; PPV: Positive predictive value; PTB: Pulmonary tuberculosis; RLS: Resource limited settings; TB: Tuberculosis; TBE: Tris-Borate EDTA; UV: Ultraviolet light; WHO: World health organization; ZN: Ziehl-Neelson.

## Competing interests

The authors declare that they have no competing interests.

## Authors’ contributions

LN, PO, MLJ, HMK, LH conceived and designed the study. LN and FAK performed the molecular assays. LN, JBS, WW, AK, MLJ and HMK analyzed the data. LN, DPK and PO wrote the manuscript. All authors read and approved the manuscript.
